# Differential expression of predisposing HLA-DQ2.5 alleles in DR5/DR7 celiac disease patients affects the pathological immune response to gluten

**DOI:** 10.1038/s41598-020-73907-2

**Published:** 2020-10-14

**Authors:** Laura Pisapia, Stefania Picascia, Federica Farina, Pasquale Barba, Carmen Gianfrani, Giovanna Del Pozzo

**Affiliations:** 1 Institute of Genetics and Biophysics, CNR, Naples, Italy; 2 Institute of Biochemistry and Cellular Biology, CNR, Naples, Italy

**Keywords:** Genetics, Immunology

## Abstract

The DR5-DQ7/DR7-DQ2 genotype is very frequent among patients affected by celiac disease (CD), in Europe. This genotype, associated to high risk of CD, carries the HLA-DQA1*05 and HLA-DQB1*02 predisposing alleles, in *trans* configuration. The alleles encode the DQ2.5 heterodimer responsible of gluten peptide presentation on the surface of antigen-presenting cells (APCs), and consequent pathogenic CD4^+^ T cell activation. We demonstrated that DR5/DR7 APCs induce an anti-gluten CD4^+^ T cell response, of comparable intensity to that observed with APCs carrying DR1/DR3 genotype, which risk alleles are in *cis* configuration. In addition, we showed that DR5/DR7 APCs from celiac patients stimulated an effector CD4^+^ T cell response higher with respect to that induced by DR5/DR7 APCs from healthy subjects. To explain these findings, we assessed the DQ2.5 RNA and protein quantity. We showed that the expression of DQA1*05 and DQB1*02 risk alleles is much higher than the expression of non-CD-associated alleles, in agreement with the previous results obtained with DR1/DR3 genotype. The differential expression of transcripts influences the quantity of DQα1*05 and DQβ1*02 chains and, as consequence, the cell surface density of DQ2.5 heterodimers. Moreover, both RNA and proteins, are more abundant in APCs from celiac patients than controls. Finally, to unravel the mechanism regulating the expression of predisposing DQA1*05 and DQB1*02 alleles, we quantified the new synthetized RNA and found that the differential expression is explained by their transcription rate. Our results confirmed that the strength of antigen-specific CD4^+^ T cell response is mainly determined by the amount of gluten in the diet and provided a new possible approach for a personalized diagnosis and for risk stratification.

## Introduction

In celiac disease (CD), the DQ2.5 haplotype, carrying HLA-DQA1*05 and HLA-DQB1*02 alleles, is the primary contributor to disease susceptibility, as it encodes the HLA-DQ2.5 heterodimer, the restriction molecule presenting gluten antigens to CD4^+^ T cells. DQA1*05 and DQB1*02 alleles may be located on the same chromosome (*cis* configuration) and in linkage disequilibrium (LD) with DRB1*03 allele, in individuals carrying the DR3-DQ2 haplotype, in the genetic asset DRB1*03-DQA1*05-DQB1*02. The DQ2.5 heterodimer is also encoded by DQA1*05 and DQB1*02 alleles located on opposite chromosomes (*trans* configuration) and in LD with DRB1*05(or DRB1*11) and DRB1*07 alleles, in individuals with the DR5/DR7 genotype^1^. Thus, the resulting HLA-DQ2.5 heterodimers, synthetized by two distinct haplotypes, differ only by two amino acids, which do not influence the functional properties. More specifically, the DQA1*05:01 allele of the DR3-DQ2 haplotype was almost identical to the DQA1*05:05 allele of the DR5-DQ7 (only differing at one single residue in the leader sequence), and the DQB1*02:01 allele of the DR3-DQ2 was almost the same of the DQB1*02:02 allele of the DR7-DQ2 haplotype (only differing at one single residue at position 135 in the membrane proximal domain)^2^. As consequence, subjects carrying DR3-DQ2 or DR5-DQ7/DR7-DQ2 genotypes express a nearly identical HLA-DQ2.5 molecule^3^. In our previous paper^4^ we demonstrated, in patients with DR1/DR3 genotype, in which CD-associated DQA1*05 and DQB1*02 alleles are in *cis* configuration, that these risk alleles are significantly more expressed than non-associated ones on the other chromosome. The preferential expression of risk alleles induces high expression of DQα1*05 and DQβ1*02 protein chains, resulting in a comparable amount of DQ2.5 heterodimers on cell surface of the antigen presenting cells (APC) in celiac patients carrying DQ2.5 genes both in homozygous and heterozygous assets.

This result supported our interest for DR5/DR7 genotype^5^ highly frequent in Southern Italy, from where our patients come. In the present work, we investigate the responsiveness of antigen-specific CD4^+^ T cells, stimulated with gliadin-pulsed APC carrying the DR5-DQ7/DR7-DQ2 genotype. Moreover, we assessed the transcripts and protein levels and investigated on the possible mechanism that might explain the risk alleles differential expression.

## Results

### B-cells with HLA DQ2.5 genes in *trans* configuration (DR5/DR7) have similar antigen presenting function of cells carrying DQ2.5 genes in *cis* configuration (DR1/DR3)

We have previously demonstrated that heterozygous APCs carrying the HLA *DQ2.5* risk alleles in cis configuration (DR1/DR3) have a comparable ability to activate the gluten-specific CD4^+^ T cells with respect to APCs homozygous for DQ2.5 genes (DR3/DR3)^4^.

**Figure 1:**
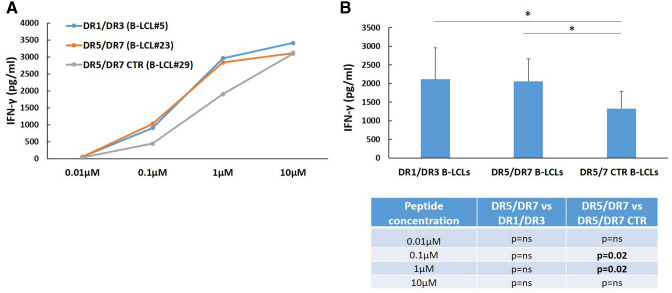
Stimulation of gliadin-reactive T cells by B-LCL with different genotypes. (**A**) Shows a representative experiment of the dose curve IFN-γ response by a DQ2.5-glia-α1,2-specific TCL obtained from celiac gut mucosa. Intestinal cells were stimulated by B-LCL#5 (from a celiac DR1/DR3), B-LCL#23 (from a celiac DR5/DR7 ), B-LCL#29 (from a control DR5/DR7). The APCs were pulsed with DQ2.5-glia-α1,2 peptide at different concentrations (0.01, 0.1, 1, 10 µM). (**B**) Shows the average of IFN-γ produced by a DQ2.5-glia-α1,2 -specific TCL at the sub-optimal DQ2.5-glia-α1,2 peptide concentration (1 µM). DR1/DR3 B-LCLs histogram represents the result of N = 5 experiments performed using as APCs celiac B-LCL#5 and B-LCL#6; DR5/DR7 B-LCLs histogram shows the result of N = 9 experiments performed using as APCs celiac B-LCL#23, #25, #26 and #27; DR5/DR7 CTR B-LCLs histogram represents the IFN-γ mean value of N = 9 experiments performed with B-LCL#29, #30, #31, #35 from healthy subjects. Table in panel B displays results of statistical analysis performed using the unpaired Student's t-test to compare B-LCLs with different genotypes, as indicated. *p < 0.05 was considered statistically significant.

In this study, the ability of heterozygous DR5/DR7 B-LCLs, from both celiacs and healthy controls, to present gliadin peptides to CD4^+^ T cells was compared to DR1/DR3 B-LCLs, in the same experimental conditions. The antigen presenting function of DR5/DR7 and DR1/DR3 B-LCLs was assessed by dosing the IFN-γ production in a T cell line generated from small intestinal mucosa of a DR5/DR7 patient previously described^6^ and highly reactive to the immunodominant gliadin peptide (DQ2.5-glia-α1,2, QLQPFPQPELPYPQPQP). DR5/DR7 B-LCL#23 (Table1), carrying the DQ2.5 genes in *trans* configuration, displayed overlapping ability to stimulate cognate TCL with respect to DR1/DR3 B-LCL#5, as shown in dose curve response to DQ2.5-glia-α1,2 peptide (Fig. 1A). Specifically, two B-LCLs, with different genotype, showed a comparable ability to induce the release of IFN-γ in response to both saturating (10 µM) and non-saturating (0.01, 0.1, 1 µM) peptide concentrations by TCL. In contrast, DR5/DR7 B-LCL#29 from non-celiac control subjects was less efficient in activating TCL at non saturating DQ2.5-glia-α1,2 peptide concentration (Fig. 1A). Figure 1B summarized all the experiments done to assess how the different chromosomal assets of DQ2.5 risk genes may impact the presentation of gliadin immunodominant peptides. Due to a limited number of expanded intestinal T cells, it was not possible to assess the APC capability of all different DQ2.5 genotypes from both celiacs and controls in the same experiment. Celiac DR1/DR3 B-LCLs (#5 and #6) were used for N = 5 experiments, celiac DR5/DR7 B-LCLs (#23, #25, #26 and #27) for N = 9 experiments and controls DR5/DR7 B-LCLs (#29, #30, #31, #35) for N = 7 experiments. DQ2.5-glia-α1,2 was used at suboptimal concentration (1 µM). No significant differences were found in the IFN-γ production when B-LCL of CD patients were used, despite the HLA genotype (p = ns).

**Table 1:** HLA-DQ haplotype and HLA-DR phenotype of subjects enrolled in the study.

APC	Diagnosis	DQA1 haplotype	DQB1 haplotype	DR phenotype
B-LCL #5	Celiac disease	*01/*05	*02/*05	DR1/DR3
B-LCL #6	Celiac disease	*01/*05	*02/*05	DR1/DR3
B-LCL #21	Celiac disease	*02/*05	*02/*03	DR5/DR7
B-LCL #22	Celiac disease	*02/*05	*02/*03	DR5/DR7
B-LCL #23	Celiac disease	*02/*05	*02/*03	DR5/DR7
B-LCL #24	Celiac disease	*02/*05	*02/*03	DR5/DR7
B-LCL #25	Celiac disease	*02/*05	*02/*03	DR5/DR7
B-LCL #26	Celiac disease	*02/*05	*02/*03	DR5/DR7
B-LCL #27	Celiac disease	*02/*05	*02/*03	DR5/DR7
B-LCL #28	Celiac disease	*02/*05	*02/*03	DR5/DR7
B-LCL #29	Control	*02/*05	*02/*03	DR5/DR7
B-LCL #30	Control	*02/*05	*02/*03	DR5/DR7
B-LCL #31	Control	*02/*05	*02/*03	DR5/DR7
B-LCL #32	Control	*02/*05	*02/*03	DR5/DR7
B-LCL #33	Control	*02/*05	*02/*03	DR5/DR7
B-LCL #34	Control	*02/*05	*02/*03	DR5/DR7
B-LCL #35	Control	*02/*05	*02/*03	DR5/DR7
B-LCL #36	Control	*02/*05	*02/*03	DR5/DR7

In addition we compared the IFN γ response of TCL stimulated with B-LCLs from celiac patient with respect to healthy control. The capability of DR5/DR7 B-LCLs from healthy control to stimulate celiac TCL resulted significantly reduced compared to celiac B-LCLs respectively of 33% (compared to DR5/DR7) and 35.9% (compared to DR1/DR3), at the sub-optimal peptide concentration of 1 µM, (Fig. 1B), (p = 0.02).

These results clearly highlight that DQ2.5 genes, either in *cis* (DR1/DR3) or in *trans* (DR5/DR7) configurations, provide to APCs high and comparable ability to stimulate antigen-specific immune response. This phenomenon is much evident with APCs from subjects with a diagnosis of CD with respect to healthy controls.

### The expression of DQα1*05 and DQβ1*02 chains is different among B-LCLs from HLA DR5/DR7 CD patients and controls

**Figure 2:**
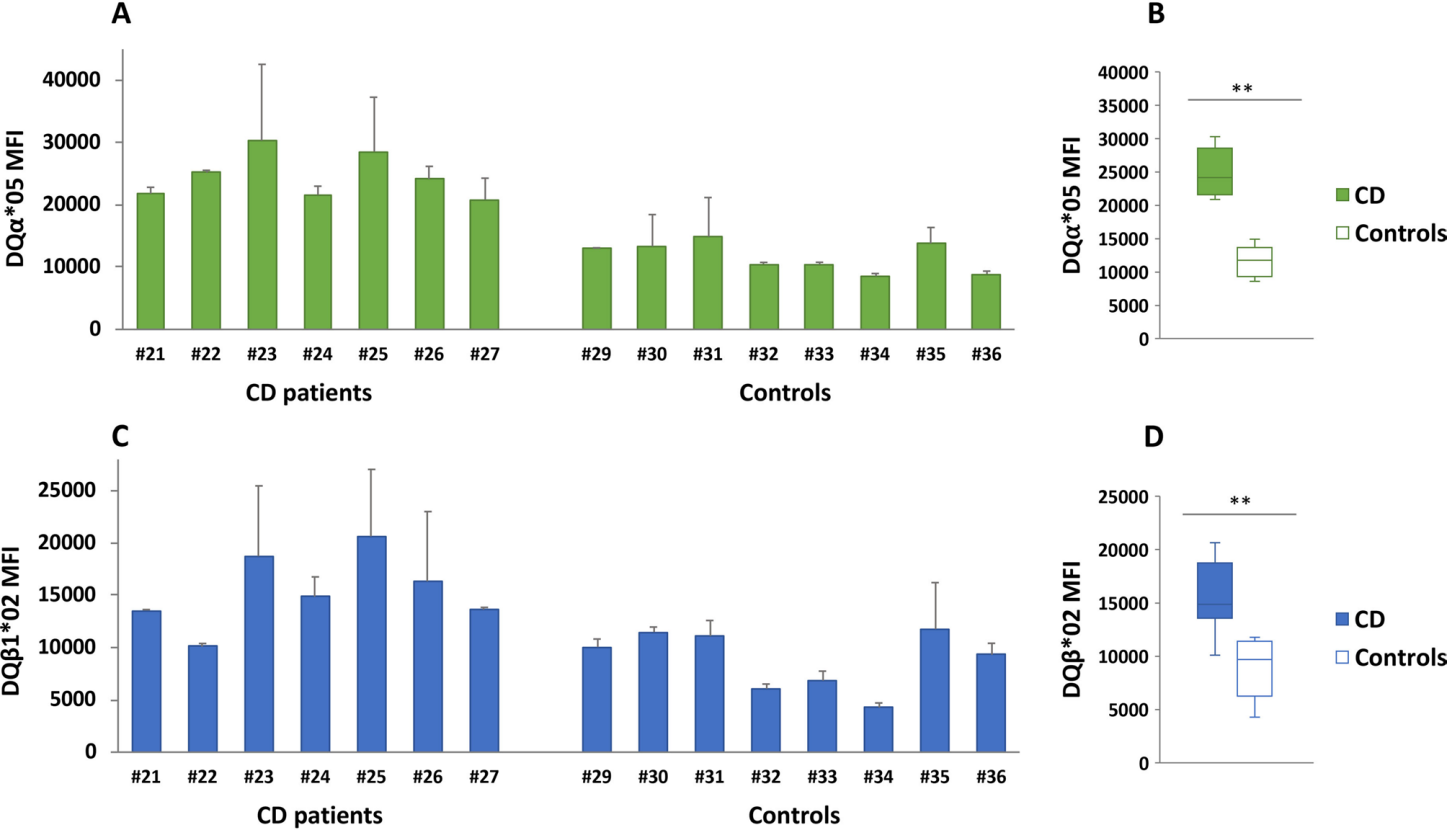
Surface expression of DQα1*05 and DQβ1*02 chains. (**A**,**C**) Show the MFI values of DQα1*05 anti-DQβ1*02 expression on B-LCL surface membrane for each single CD patient (left) and control (right). (**B**,**D**) show the MFI mean of DQα1*05 anti-DQβ1*02 chain staining in B-LCLs from all CD patients and healthy controls. (*p < 0.05, **p < 0.005).

To explain the difference in the ability of DR5/DR7 B-LCLs from celiac patients and controls, we quantified the amount of DQα1*05 and DQβ1*02 protein chains forming the DQ2.5 heterodimeric molecules. The evaluation of the expression level of HLA DQ2.5 heterodimers was assessed by specific monoclonal antibodies against DQα1*05 and DQβ1*02 proteins, since antibodies directed against the DQ2.5 heterodimer are not available. The expression of DQα1*05 chain on B-LCL from each patient and control subjects is shown in Fig. 2A, while Fig. 2B showed the average variation in the Mean Fluorescent Intensity (MFI) value observed between the two groups. Similarly, the expression of DQβ1*02 molecule on B-LCL from DR5/DR7 patients and control subjects was shown in Fig. 2C and the statistical comparison of MFI between two group was reported in Fig. 2D. Our results clearly demonstrated a greater surface expression of DQα1*05 and DQβ1*02 chains in CD patients with respect to healthy controls carrying the DR5/DR7 genotype. These results explain the higher gliadin-specific CD4^+^ T cell activation when stimulated by DR5/DR7 APCs from celiacs with respect to controls (Fig. 1).

### CD-associated DQA1*05 and DQB1*02 alleles are more expressed in B-LCL carrying DR5-DR7 genotype

**Figure 3:**
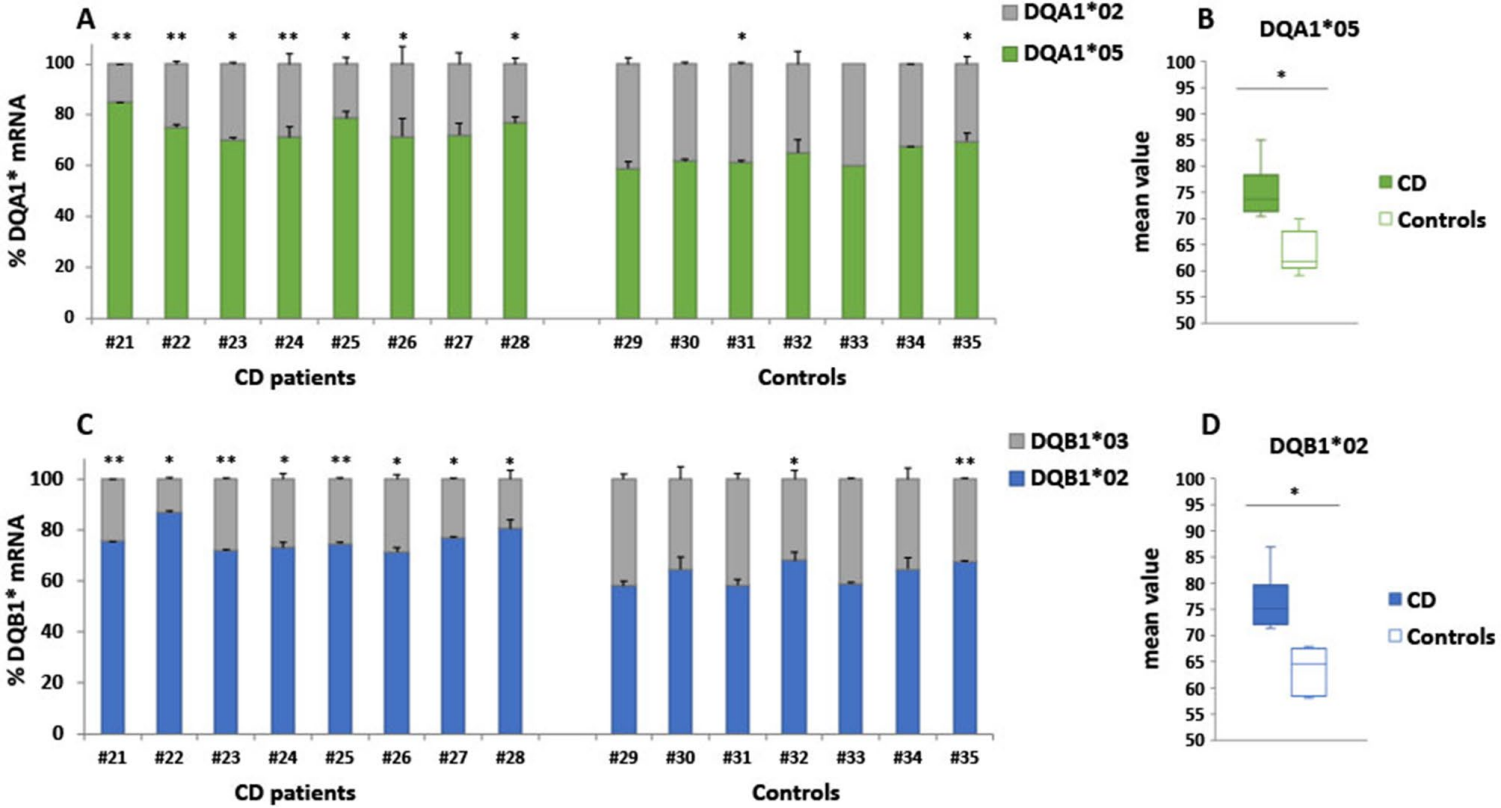
Expression level of DQB1* and DQA1* genes in HLA DR5/DR7 B cell lines. The gene expression is showed as percentages of the total *DQA1* and *DQB1* transcripts for each B-LCL. (**A**,**C**) show the expression of *DQA1* alleles in B-LCL from patients (left) and healthy controls (right); (**B**,**D**) show the mean percentages of DQA1*05 and DQB1*02 mRNA measured in B-LCLs from all CD patients and healthy controls analyzed (*p < 0.05, ** p < 0.005).

To explain functional data, we measured the mRNA amount of CD-associated DQA1*05 and DQB1*02 alleles when they are in *trans* configuration with respect to the RNA amount of alleles not associated with the disease. DQA1* and DQB1* transcripts were quantified by qRT-PCR in B-LCLs with DR5/DR7 genotype from 8 CD patients (#21 to #28 B-LCL) and 7 healthy subjects (#29 to #36 B-LCL) (Table 1). The amount of mRNA encoded by each allele was expressed as percentage of total DQA1* transcripts (Fig. 3A) and as percentage of total DQB1* transcripts (Fig. 3C). We observed that the mean percentage of DQA1*05 mRNA was 75%, significantly higher than the DQA1*02 mRNA mean percentage (Fig. 3A). Similarly, the mean amount of DQB1*02 mRNA was 76%, significantly greater than DQB1*03 mRNA (Fig. 3C). However, the differences between the messengers transcribed by the two alleles were lower when we analyzed both DQA1* and DQB1* genes in B-LCL from the healthy subjects with the same genotype (DR5/DR7). In fact, we observed a significant difference when we compared the mean percentages obtained for DQA1*05 mRNA in B-LCL from patients (75%) and controls (64%) (Fig. 3B), as well as when we compared the mean percentages obtained for DQB1*02 mRNA in patient (76%) and control (63%) cells (Fig. 3D). These differences correspond to a 7–8% of increment in the mean value of expression. In conclusion, both DQA1*05 and DQB1*02 risk alleles, when located in *trans* configuration, are more expressed than DQA1* and DQB1* alleles not CD-associated on the other chromosome, according to our previous results obtained on DR3DQ2 positive CD and control subjects^4^.

### The differential expression of risk alleles is determined by de novo transcription

**Figure 4:**
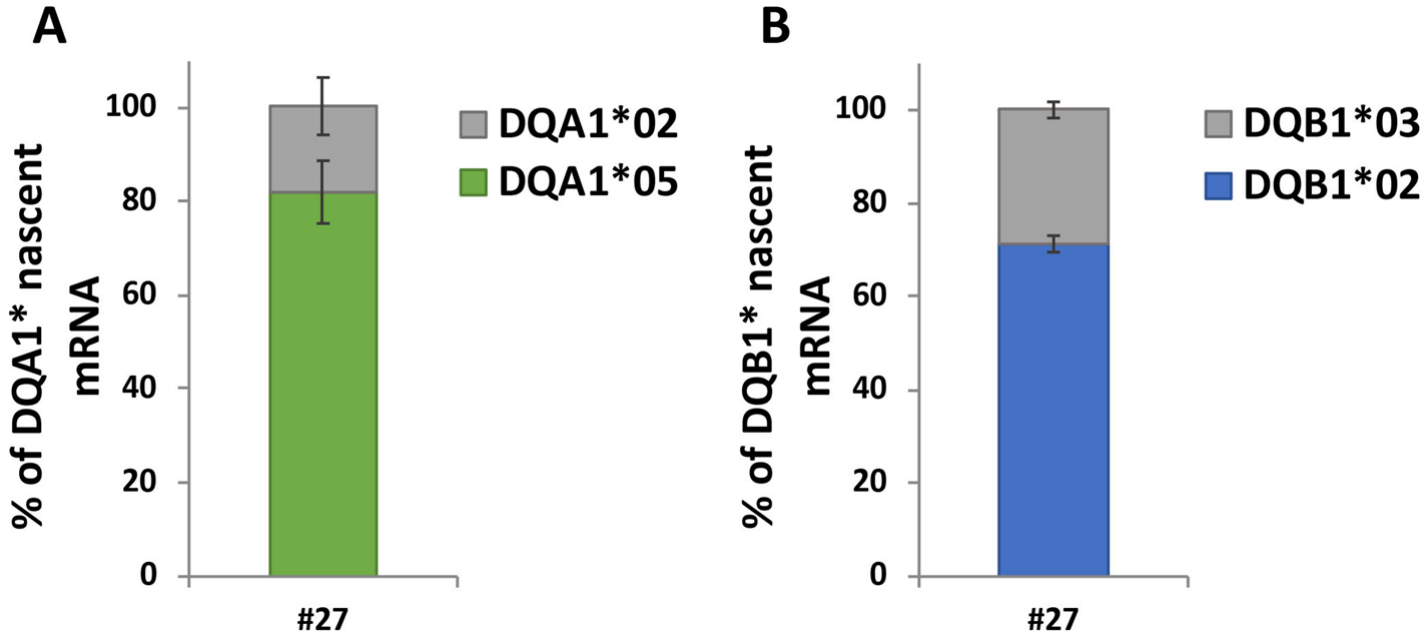
Relative expression of the newly synthetized DQB1* and DQA1* transcripts. The amount of new transcribed DQB1* and DQA1* mRNA in B-LCL#27 is shown as percentages of the total transcript. The relative expression of DQA1*05 versus DQA1*02 and DQB1*02 versus DQB1*03 mRNA is reported in panel A and B, respectively.

In order to unravel the mechanism responsible of differential alleles expression we used click- iT chemistry^7^ able to monitor nascent transcripts. Indeed, we investigated if the differential expression of DQA1*05 and DQB1*02 alleles with respect to DQA1*02 and DQB1*03 might be determined by a different rate of transcription. We labeled growing B-LCL#27 with a uridine analog, the 5-ethynyluridine (EU) that is incorporated into the nascent RNA allowing us to assess the rate of transcription of CD associated DQA1*05 and DQB1*02 alleles with respect to non-CD associated DQA1*02 and DQB1*03. After an overnight incubation, cells are harvested and EU-labeled RNA, deriving from de novo transcription, is prepared and used to synthetize cDNA to quantify the RNA transcribed by each allele by qRT-PCR. As reported in Fig. 4A, the new synthetized HLA-DQA1 mRNA is mainly transcribed by DQA1*05 with respect to DQA1*02 allele (82% versus 18%). Similarly, the new transcribed DQB1*02 mRNA is higher than DQB1*03 mRNA (71% versus 29%, Fig. 4B). Our results clearly demonstrated a greater rate of transcription of CD risk alleles either in *cis* (data not shown) or in *trans* (Fig. 4) configurations and explain the different amount of DQA1*05 and DQA1*02 RNA found in DR5/DR7 B-LCLs.

## Discussion

The CD risk alleles HLA DQA1*05 and DQB1*02 encode the HLA DQ2.5 molecule either when they are in *cis* (DR3-DQ2 haplotype), or in *trans* configuration (DR5-DQ7/DR7-DQ2 genotype). Both are associated to high risk for CD. The risk to develop CD has been associated to the ability of DQ2.5 molecule to present full repertoire of gliadin-derived antigenic peptides to CD4^+^ T cells^8^. In our previous work, we demonstrated that the APCs carrying the risk genes in *cis* configuration, both homozygous (DR3/DR3) and heterozygous (DR1/DR3) for DQ2.5, have the same stimulatory capability on gluten-reactive CD4^+^ T cells^4^. This result was explained by the high expression of DQA1*05 and DQB1*02 alleles also in heterozygous (DR1/DR3) genotype and, as a consequence, by the great density of DQ2.5 heterodimers on APC membrane surface. In the present work, we expanded our analysis to APCs carrying the predisposing alleles in *trans* configuration, as the DR5/DR7 genotype is very frequent in Southern Europe and associated to high risk. We showed that APCs with DR5/DR7 genotype induces an anti-gluten CD4^+^ T cell stimulation as strong as APCs carrying DR1/DR3 from celiac patients, and that the magnitude of CD4^+^ T cells response depends on the antigen concentration. Moreover, we observed that the strength of CD4^+^ T cell activation is lower when stimulated by DR5/DR7 APC from healthy subjects. This result was confirmed by the reduced expression of DQα1*05 and DQβ1*02 surface chains in controls that impacts on the density of heterodimeric DQ2.5 molecule. When we measured the expression of both DQA1*05 and DQB1*02 risk alleles, we found a higher amount of RNA with respect to non-disease associated DQA1*02 and DQB1*03 transcripts. The differential expression of alleles was much stronger in APC from celiac patients that non-affected subjects, although the latter showed an RNA quantity higher than 50%. To gain information regarding the molecular mechanism that induces the differential expression of risk alleles and to formulate a functional hypothesis, we investigated on the transcription rate. Through click-iT chemistry, monitoring the incorporation of an analog of uridine during transcription, we quantified the new synthetized DQA1 and DQB1 mRNAs. This experiment demonstrated that the great expression of risk alleles DQA1*05 and DQB1*02 is mainly due to a difference in the *transcription rate* with respect to DQA1*02 and DQB1*03 alleles non-associated to the pathology. These findings and others, relative to Type 1 diabetes^9^ and multiple sclerosis^10^, suggested that the high transcription is typical of HLA-*DQ* or HLA-*DR* risk alleles associated to autoimmune diseases, regardless of the haplotype to which they belong. Several papers, in the recent literature, demonstrated the relationship between genomic variants in the intergenic regions across the MHC locus, and the expression of risk alleles predisposing various autoimmune diseases^11–13^. The correlation between eQTL and MHC haplotype sequence variation has been assessed^14^, as well as the differential expression of *DQA1* and *DQB1* alleles in heterozygous CD4^+^ T cells^15^, depending on lymphocytes activation. In addition, we might also speculate differences in the regulative function of genes and/or factors included in the upstream activation pathways of risk alleles that, in pathological conditions, might cause stronger promoter activity. We propose that the high transcription rate should be attributed to only one allele, encoding alfa or beta chain, because we demonstrated a coordinate regulation of two genes encoding the HLA class II heterodimer^9^.

In conclusion the preferential expression of risk alleles impacts on DQ2.5 surface density and on the efficacy of gliadin antigen presentation. The risk to develop CD is associated to the threshold of CD4^+^ T cells activation, strictly dependent on the amount of gluten immunogenic peptides exposed by intestinal T lymphocytes. This conclusion was proven by the comparable strength of antigen-specific CD4^+^ T cells activation when stimulated by antigen-pulsed DR3/DR3 homozygous or heterozygous DR3/DR1 and DR5/DR7 APCs. All APCs, regardless their haplotypes, stimulated the gliadin-specific CD4^+^ T cells reaching the plateau of activation, thanks to the large amount of HLA-DQ2.5-gluten epitopes complex presented on APC membrane surface. In addition, our data demonstrated that the expression level of predisposing alleles, found much higher in CeD patients with respect to healthy controls, might be quantified with a Δ value representing the difference in the DQ expression genes between the two groups of subjects. This opens the possibility to further stratify the CD genetic risk and to support an early disease diagnosis. However the high risk associated to DR3/DR3 patients highlighted by many papers might be explained by the contribute of other loci accounting for an additional 18% of genetic heritability^16^ in addition to the role of the B*0801 gene of the HLA class I region in LD with DR3-DQ2.5 genes^17^.

Our molecular data, demonstrating the high risk associated to DR5/DR7 genotype, are fortified and supported by genetic studies demonstrating that DQA1*05:01 and DQB1*02:01 alleles are the primary contributors to CD susceptibility. These studies are based on the availability of GWAS^18^ data and on several HLA imputation programs that define genetic models of association between classical HLA alleles and autoimmune diseases. Lenz et al.^19^ using the SNP2HLA^20^ imputation algorithm demonstrated that, in addition to the additive model, in which the risk associated to a specific haplotype depends on the dosage effect or on the sum of risk for each allele, there is a non-additive model in which the synergistic interactions between different haplotypes confer a great disease risk. This genetic point of view confirmed the strong celiac disease risk associated with DR5/DR7 genotype, herein analyzed.

## Conclusions

The molecular data we presented highlight the pivotal role that mRNA has in the risk stratification and opens to a new approach in supporting an early diagnosis of CD and autoimmune disorders on the assessment of DQ2.5 gene expression.

## Methods

### Antigen presenting cells

The EBV-transformed, B lymphoblastoid cell lines (B-LCL) were established from PBMCs of celiac patients or non-celiac donors by infection with a culture medium of Marmoset cells^21^, previously titrated for virulence, in the presence of cyclosporine A at 0.5 mg/ml. All CD volunteers and healthy controls, recruited as a source of APCs, as well as for the generation of gluten reactive T cell lines and clones, were typed for DQA1, DQB1 and DRB1 haplotypes by PCR using the following HLA typing kits: AllSet Gold SSP HLA-DR Low Res kit, AllSet Gold SSP HLA-DQ Low Res kit and DQA1 SSP UniTray kit, all from Invitrogen (Life Technologies). The haplotypes of the B-LCL are reported in Table 1.

### T cell lines generation and functional tests

A T cell line (TCL) was previously established from mucosal explants of a HLA DQ2.5 CD patient with DR5/DR7 genotype and was used in functional assay to assess the capability of B-LCLs to present gliadin epitopes^6^. Briefly, mucosal explants were digested with collagenase A (1 mg/ml), and intestinal cells (3–5 × 10^5^) were incubated with irradiated autologous PBMCs (1.5 × 10^6^) and TG2-treated (deamidated) gliadin (50 µg/ml), in complete medium (X-Vivo 15 medium supplemented with 5% AB-pooled human serum and antibiotics, Lonza). Thereafter, long term cultures were obtained by stimulating growing CD4^+^ T cells with (1.5 × 10^6^) irradiated allogenic PBMCs cells and phytohemagglutinin (PHA) (0.5 µg/ml). When in resting phase, the TCL was assayed in response to the cognate gliadin DQ2.5-glia-α1,2 epitope (QLQPFPQPELPYPQPQP, provided by CASLO ApS, Kongens Lyngby, Denmark). Briefly, TCL cells (3 × 10^4^) were co-incubated with B-LCL (1 × 10^5^) derived from CD patients (DR5/DR7 and DR1/DR3) and healthy controls (DR5/DR7), in presence of DQ2.5-glia-α1,2 peptide. In peptide dose–response experiments, B-LCLs were pulsed for 4 h at 37 °C with peptide using escalating concentrations (from 0.01 to 10 µM) in 96-well round bottom plates (200 µL volume), all in duplicates. After the incubation, B-LCLs were washed to remove unbound peptides before adding TCL. Cells were incubated at 37 °C in complete medium (X-Vivo enriched with 5% human serum) in U-bottom 96-well plates. Cell supernatants (50 µL) were collected after 48 h for the evaluation of INF-γ by standard sandwich ELISA procedure.

### Monoclonal antibodies and flow cytometry analysis

B-LCLs were harvested at sub-confluence and were suspended at 10^6^ cells/ml in ice cold PBS, 10% FCS and 1% NaN3. Then, 100 ml of cell suspension was plated in a 96 U-bottom plate and labelled with 1 μg/ml of primary or isotypes control monoclonal antibodies, or with 10 μl of the hybridoma supernatant, previously titrated. The cells were incubated at 4 °C in the dark for 30 min, washed and thereafter labelled with secondary antibodies at a final concentration of 10 μg/ml in 3% BSA/PBS for an additional 30 min at 4 °C in the dark. The primary monoclonal antibodies used to reveal the cell surface HLA DQ expression were: SFR20-DQa5, a rat anti-HLADQA1*05 purified from a hybridoma supernatant, kindly provided by Prof Radka^22^; 2.12E11, a murine anti-HLA-DQB1*02, kindly provided by Prof Sollid^23^. Fluorochrome conjugated anti-rat IgG (-PE) and anti-mouse IgG (-FITC) were used as secondary antibodies.

All phenotypes were analysed with FACSCanto II system and elaborated using the DIVA software (BD Biosciences).

### Steady-state and nascent and mRNA quantization

Total RNA from B-LCL was prepared with the Aurum Total RNA kit (Bio-Rad), and 1 μg of RNA was used for reverse transcriptase reactions, performed using an iScript cDNA Synthesis kit (Bio-Rad).The amount of specific transcripts was measured by qRT-PCR using the Quanti Tect SYBR Green PCR Kit (Bio-Rad) through the DNA Engine Opticon Real-Time PCR Detection System (Bio-Rad). Each reaction was run in triplicate in the presence of 0.2 mM primers synthesized by Eurofins, and each experiment was performed four times^4^. The primer sequences are reported in Table 2. The relative amount of specific transcripts was calculated by the comparative cycle threshold method^24^ and β-actin transcript was used for normalization.

**Table 2:** Primers used for qPCR.

Genes	Primers	Sequences
GAPDH	GAPDH-F	GAAGGTGAAGGTCGGAGTC
	GAPDH-R	GAAGATGGTGATGGGATTTC
β-ACTIN	ACTβ-F	TCATGAAGTGTGACGTTGACA
	ACTβ-R	CCTAGAAGCATTTGCGGTGCAC
HLA-DQA1*05	DQA1*05-F	TGGTGTTTGCCTGTTCTCAGAC
	DQA1*R	GGAGACTTGGAAAACACTGTGACC
HLA-DQA1*02	DQA1*02-F	AAGTTGCCTCTGTTCCACAGAC
	DQA1*-R	GGAGACTTGGAAAACACTGTGACC
HLA-DQB1*02	DQB1*02-F	TCTTGTGAGCAGAAGCATCT
	DQB1*-R	CAGGATCTGGAAGGTCCAGT
HLA-DQB1*03	DQB1*03-F	CGGAGTTGGACACGGTGTGC
	DQB1*-R	CAGGATCTGGAAGGTCCAGT

The newly synthesized RNA transcripts were captured by Click-iT Nascent RNA Capture Kit (Thermo Fisher Scientific) according to manufacturer’s instructions. Briefly, B-LCLs, seeded at 50% confluency, were labelled with 0.2 mM ethynyl uridine (EU) and incubated at 37 °C for 16 h. Total RNA was prepared with TRIzol reagent (Life Technologies). The EU-labeled RNAs were biotinylated with 0.5 mM biotin azide in Click-iT reaction buffer. The biotinylated RNAs were precipitated and resuspended in distilled water. 0.5 µg of purified RNA was bound to 25 μl of Dynabeads MyOne Streptavidin T1 magnetic beads in Click-iT RNA binding buffer. The RNA captured on the beads was used as template for cDNA synthesis. Reverse transcription was performed using the SuperScript VILO cDNA Synthesis Kit Invitrogen (Thermo Fisher Scientific) following the manufacturer's instructions. The amount of specific transcripts was measured by qRT-PCR using Sso Advanced SYBR Green PCR Kit (Bio-Rad). The apparatus and primers used were the same described above.

### Statistical analysis

All results shown are the mean of at least three independent experiments. Statistical analysis was performed using the unpaired Student's t-test with two-tailed distribution and assuming two samples equal variance parameters. In the figures, a single asterisk corresponds to p < 0.05 and double asterisks correspond to p < 0.01.

### Ethical statements

We declare that: (1) this study including B-LCLs and TCLs from celiac patients and healthy subjects was approved by the ethical committee of Department of Pediatrics University Federico II of Naples, Italy (Register 343/17 dated 01/30/2018); (2) this study was conducted in accordance with the Declaration of Helsinki; (3) all participants gave their written informed consent for inclusion in this study.

## References

[1] Sollid LM, Qiao SW, Anderson RP, Gianfrani C, Koning F (2012). Nomenclature and listing of celiac disease relevant gluten T-cell epitopes restricted by HLA-DQ molecules. Immunogenetics.

[2] Sollid LM (2017). The roles of MHC class II genes and post-translational modification in celiac disease. Immunogenetics.

[3] Margaritte-Jeannin P (2004). HLA-DQ relative risks for coeliac disease in European populations: A study of the European Genetics Cluster on Coeliac Disease. Tissue Antigens.

[4] Pisapia L (2016). HLA-DQ2.5 genes associated with celiac disease risk are preferentially expressed with respect to non-predisposing HLA genes: Implication for anti-gluten T cell response. J. Authoimmunity.

[5] Delgado JF (2014). Paediatric celiac patients carrying the HLA-DR7-DQ2 and HLA-DR3-DQ2 haplotypes display small clinical differences. Acta Paediatr. Int. J. Paediatr..

[6] Camarca A (2017). Gliadin-reactive T cells in Italian children from preventCD cohort at high risk of celiac disease. Pediatr. Allergy Immunol..

[7] Lu XM (2017). Nascent RNA sequencing reveals mechanisms of gene regulation in the human malaria parasite *Plasmodium falciparum*. Nucleic Acids Res..

[8] Scherf KA (2020). Recent progress and recommendations on celiac disease from the working group on prolamin analysis and toxicity. Front. Nutr..

[9] Farina F  (2019). HLA-DQA1 and HLA-DQB1 alleles, conferring susceptibility to celiac disease and type 1 diabetes, are more expressed than non-predisposing alleles and are coordinately regulated. Cells.

[10] Pisapia L (2019). The HLA-DRB1 risk alleles for multiple sclerosis are differentially expressed in blood cells of patients from Southern Italy. Int. J. Immunogenet..

[11] Gianfrani C, Pisapia L, Picascia S, Strazzullo M, Del Pozzo G (2018). Expression level of risk genes of MHC class II is a susceptibility factor for autoimmunity: New insights. J. Autoimmun..

[12] Raj P (2016). Regulatory polymorphisms modulate the expression of HLA class II molecules and promote autoimmunity. eLife.

[13] Cavalli G (2015). MHC class II super-enhancer increases surface expression of HLA-DR and HLA-DQ and affects cytokine production in autoimmune vitiligo. Proc. Natl. Acad. Sci..

[14] Star A, Genetics M (2017). Unique allelic eQTL clusters in human MHC haplotypes. G3 Genes Genomes Genet..

[15] Gutierrez-Arcelus M (2020). Allele-specific expression changes dynamically during T cell activation in HLA and other autoimmune loci. Nat. Genet..

[16] Gutierrez-Achury J (2015). Fine mapping in the MHC region accounts for 18% additional genetic risk for celiac disease. Nat. Genet..

[17] Mazzarella G (2008). Gliadin activates HLA class I-restricted CD8+ T cells in celiac disease intestinal mucosa and induces the enterocyte apoptosis. Gastroenterology.

[18] Trynka G (2012). Dense genotyping identifies and localizes multiple common and rare variant association signals in celiac disease. Nat. Genet..

[19] Lenz TL (2015). Widespread non-additive and interaction effects within HLA loci modulate the risk of autoimmune diseases. Nat. Genet..

[20] Jia X (2013). Imputing amino acid polymorphisms in human leukocyte antigens. PLoS ONE.

[21] Cho YG (2001). An Epstein–Barr-related herpesvirus from marmoset lymphomas. Proc. Natl. Acad. Sci. USA..

[22] Amar A (1987). Characterization of specific HLA-DQ alpha allospecificities by genomic, biochemical, and serologic analysis. J. Immunol..

[23] Viken HD (1995). Characterization of an HLA-DQ2-specific monoclonal antibody. Influence of amino acid substitutions in DQβ 1*0202. Hum. Immunol..

[24] Livak KJ, Schmittgen TD (2001). Analysis of relative gene expression data using real-time quantitative PCR and the 2−ΔΔCT method. Methods.

